# Solving the elusiveness of word meanings: two arguments for a continuous meaning space for language

**DOI:** 10.3389/frai.2023.1025293

**Published:** 2023-06-19

**Authors:** Maria M. Piñango

**Affiliations:** Department of Linguistics and Interdepartmental Neuroscience Program, Yale University, New Haven, CT, United States

**Keywords:** word meaning, semantic memory, episodic memory, language and cognition, lexico-conceptual structure, meaning computation, conceptual semantics

## Abstract

I explore the hypothesis that the experience of meaning discreteness when we think about the “meaning” of a word is a “communicative” illusion. The illusion is created by processing-contextual constraints that impose disambiguation on the semantic input making salient a specific interpretation within a conceptual space that is otherwise continuous. It is this salience that we experience as discreteness. The understanding of word meaning as non-discrete raises the question of what is context; what are the mechanisms of constraint that it imposes and what is the nature of the conceptual space with which pronunciations (i.e., visual/oral signs) associate themselves. I address these questions by leveraging an algebraic continuous system for word meaning that is itself constrained by two fundamental parameters: control-asymmetry and connectedness. I evaluate this model by meeting two challenges to word meaning discreteness (1) cases where the same pronunciation is associated with multiple senses that are nonetheless interdependent, e.g., English “smoke,” and (2) cases where the same pronunciation is associated with a family of meanings, minimally distinct from each other organized as a “cline,” e.g., English “have.” These cases are not marginal–they are ubiquitous in languages across the world. Any model that captures them is accounting for the meaning system for language. At the heart of the argumentation is the demonstration of how the parameterized space naturally organizes these kinds of cases without appeal for further categorization or segmentation of any kind. From this, I conclude that discreteness in word meaning is epiphenomenal: it is the experience of salience produced by contextual constraints. And that this is possible because, by and large, every time that we become consciously aware of the conceptual structure associated with a pronunciation, i.e., its meaning, we do so under real-time processing conditions which are biased toward producing a specific interpretation in reference to a specific situation in the world. Supporting it is a parameterized space that gives rise to lexico-conceptual representations: generalized algebraic structures necessary for the identification, processing, and encoding of an individual's understanding of the world.

## 1. Introduction

**Figure 1:**
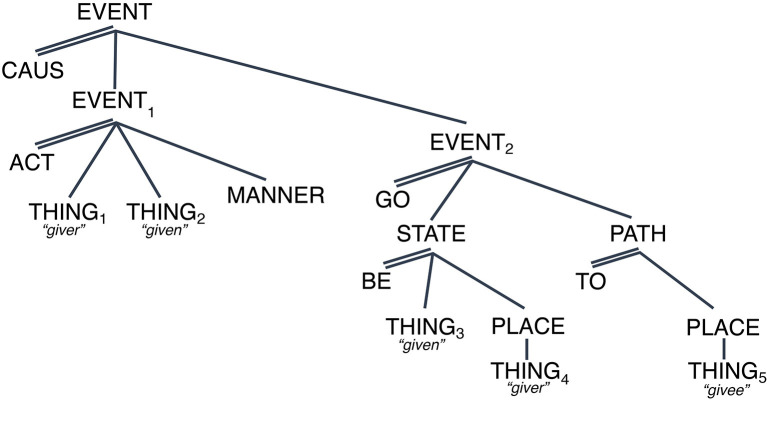
LCS of English [giv]: [CAUS [ACT (THING_1_, THING_2_)]_EVEN_T__1__, [GO [BE ((THING_3_), PLACE (THING_4_)], [PATH [to (THING_5_)]]]_EVEN_T__2__]_EVENT_.

Most linguistic meaning comprehension occurs flawlessly, supporting a compositional analysis based on pre-specified parts. These parts are not only the meanings associated with words but also with any other morphophonological or morphosyntactic element e.g., bound stems and affixes. The order in which the “parts” are put together also contributes to the meaning of the resulting segment. Consider the lexico-conceptual representation associated with English [gıv] “give” in Figure 1 below:^1^,^2^

In Figure 1 I use the geometrical representation for ease of exposition. This is the most common equivalent: [CAUS [ACT (THING_1_, THING_2_)]_EVEN_T__1__ [GO [BE ((THING_3_), PLACE (THING_4_)], [PATH_to_ (THING_5_)]]_EVEN_T__2__]_EVENT_. Both representations simply state that English [gıv] minimally refers to a causal event involving two (sub) events: in EVENT_1_, THING_1_ the *agent*, carries out an ACTion on THING_2_ the result of which is EVENT_2_: THING_3_, the *theme* (coreferent with THING_2_), which undergoes a spatial change from THING_4_, the *source* (coreferent with THING_1_), onto THING_5_, the *goal*. The syntactic representation for the sentence “The woman gave the ball to the girl” [NP [V NP PP]] closely “tracks” the conceptual structure. Consider now the sentences in (1) below:

(1) (a) The woman gave the girl the ball → [NP [V NP NP]_VP_]_S_
(b) ^#^The woman gave the ball the girl → [NP [V NP NP]_VP_]_S_

Both 1(a) and 1(b) contain the same word meaning-pronunciation pairings and identical syntactic categories and configurations, but their meanings differ:

$\begin{array}{l} \left(2)\;[{\text{NP}}_{\text{1=4}}[{\text{V}}_{\text{give}}\text{N}{\text{P}}_{5}\text{N}{\text{P}}_{\text{2=}\;\text{3}}]\right]\\ \to \text{LCS}=[\text{CAUS}\;{\left[\text{ACT}\left(\text{woma}{\text{n}}_{1}\;\text{bal}{\text{l}}_{2}\right)\right]}_{\text{EVENT},}[\text{GO}\;{\left[\text{BE}\;\left(\text{bal}{\text{l}}_{3}\right)\text{PLACE}\left(\text{woma}{\text{n}}_{4}\right)\right]}_{\text{STATE},}\\ \;[\text{PATH}[\text{TO}[\left(\text{gir}{\text{l}}_{5}\right)]]\;]\text{EVENT}{]}_{\text{EVENT}} \end{array}$

$\begin{array}{l} \left(3)\;[{\text{NP}}_{\text{1=4}}[\text{VNP}{\text{\$}}_{\;\text{2=3}}\;\text{N}{\text{P}}_{5}]\right]\\ \to^{\#}[\text{LCS}=[\text{CAUS}\;{\left[\text{ACT}\left(\text{woma}{\text{n}}_{1}\;\text{gir}{\text{l}}_{2}\right)\right]}_{\text{EVENT},}[\text{GO}\;{\left[\text{BE}\;\left(\text{gir}{\text{l}}_{3}\right)\text{PLACE}\left(\text{woma}{\text{n}}_{4}\right)\right]}_{\text{STATE},}\\ \;[\text{PATH}\;[\text{TO}[\left(\text{bal}{\text{l}}_{5}\right)]]\;]\text{EVENT}{]}_{\text{EVENT}} \end{array}$

What changes between (2) and (3) is the *linking that the syntactic structure dictates* as shown by the shared subscripts. Example (2) illustrates the most plausible interpretation. Example (3) shows the alternative linking which yields a less plausible interpretation (implausibility signaled with #). As examples (2) and (3) above show then, the interaction of word meanings and ways of composition appears to be the result of the meanings of the words in the sentence and the way they are organized within the sentence, manifested here in the linking with conceptual structure; an idea normally expressed as the principle of compositionality.^3^

### 1.1. The problem: compositionality does not exhaustively lead to discrete word meanings

According to the above definition, compositionality would suggest that if we were to ask about the meanings of the composing words—the semantic building blocks of the sentence—we could turn the process around by factoring out the linking to morphosyntactic structure and “distribute” the remaining meaning of the sentence into those building blocks. For many linguistic constructions this process does not lead to the expected set of word meanings.

Indeed, the reality of the relation between sentence meaning and the meaning of the words that compose it is less direct than one would expect. Three kinds of cases reveal this: meaning underspecification, meaning ambiguity, and meaning “dislocation.” (1) Meaning underspecification is observed when the composition of word meanings fails to express the sentential meaning observed. This is evidenced in cases of so-called “enriched composition” (e.g., Pustejovsky, 1995; Jackendoff, 1997; Piñango et al., 1999, 2006; McElree et al., 2001; Lapata et al., 2003; Wiese and Maling, 2005; Pylkkänen and McElree, 2007; Deo and Piñango, 2011; Katsika et al., 2012; Lai et al., 2017a,b, 2023; Piñango, 2019). In the sentence “The rabbit jumped for a long time in the garden,” the preferred interpretation is that the rabbit jumped *repeatedly*. Yet, the meaning of repetition is nowhere in the sentence. (2) Meaning ambiguity is observed in situations where the same pronunciation leads to more than one interpretation that may be conceptually unrelated, e.g., homonymy, and sometimes related, e.g., polysemy (e.g., Swinney, 1979; MacDonald et al., 1994; Pustejovsky, 1995). Such situations also give rise to the “meaning” vs. “sense” distinction distinguishable by content that is entailed, meaning, from content that is better viewed as implied, sense (e.g., Frazier and Rayner, 1990). One lesser discussed case of meaning ambiguity refers to the composition of the same “word” that gives rise to different yet not unrelated interpretations depending on the other words that it combines with. In the sentence “This summer, Taylor decided to grow tomatoes and a mustache,” the sense of *grow* that emerges with “tomatoes” differs from the one that emerges with “mustache” yet both ‘senses' appear connectable to a unified meaning (e.g., See Pustejovsky, 1995 for an extensive discussion of this issue). Finally, (3) meaning “dislocation” is observed in cases where the meaning of the parts is minimally relatable to the meaning of the whole. This is normally observed in so-called idiomatic expressions. In the phrase “to lose face” as in “Sam did not want to lose face in front of their friends,” e.g., to appear less credible to others at a specific point in time, very little of the meaning of the expression is seen as emerging from the meaning of the meanings for “lose” and for “face,” yet it is not the case that either of those meanings is completely disconnected from the idiomatic interpretation.^4^

We thus find ourselves in a tension: on the one hand, when we hear/see a pronunciation of a lexical item in the context of a sentence, we get an interpretation that is uniquely associated with that pronunciation based on how it relates to the meaning of the sentence but, on the other hand, the meaning of the sentence is neither exhausted by the meanings identified for those pronunciations that are contained in them nor do those meanings always succeed in predicting it. Altogether what the above three types of cases suggest is the difficulty in predictably and exhaustively associating a lexical pronunciation with *a* meaning *in the absence of additional context*. These two properties, predictability and exhaustivity, are two desiderata of word meaning discreteness, yet what we observe is meaning *interdependence* and *unboundedness*.^5^

We conclude that a view of word meanings that calls for them to be discrete and encapsulated in the language system in any way, as implied for example by the Saussurian sign-signifier pairing, is not *prima facie* supported by the evidence. How then should we understand word meanings? What gives rise to the experience of discreteness? How can we explain linguistic meaning composition based on word meaning interdependence and unboundedness?

The solution that I explore here demands that we assume that lexicalization—the process of generating an association of pronunciation structure, morphological structure and syntactic structure with meaning—takes place not as the result of isolating discrete concepts but as a process of systematically connecting pronunciations to conceptual content that is itself organized in a continuous mental space. On this view, “discreteness” amounts to the sense of salience that results from directed attention to a segment in an LCS, guided by local context in real-time, as sentence comprehension unfolds. This makes discreteness a by-product of the process of comprehension, not a feature of the lexico-conceptual system itself. From this perspective, context is a mental space informed by multiple kinds of constraints including plausibility considerations, and, as I argue here, organized along two parameters: control-asymmetry and connectedness.

Before discussing the specifics of the continuous space, however, I will explore two cases that illustrate the two fundamental challenges to discreteness as a property of word meanings that any model of conceptual structure for language must address. The challenges are (1) the interdependence of distinct word “senses” (interdependence) and (2) the lack of inherent segmentation between word meanings (unboundedness). The first will be discussed in the context of the meaning of English [smə**℧**k] “smoke” and the second will be discussed in the context of the meaning of English [hæv] “have.”

#### 1.1.1. Challenge 1. How to account for distinct word “senses” when their interdependence is entailed: the case of English [smə℧k]

Consider the meaning of English [smə℧k] in the sentences below (most of them extracted from Jackendoff, 2012 Ch 6):^6^

(4) The fire gave off a lot of **sm**ə**℧k**_**Noun**_
[smə**℧**k] → the gas-based substance

(5) The fires were reported to **sm**ə**℧k**_**Verb**_ a lot in California.
[smə**℧**k] → the production of the gas-based substance

(6) The view of chimneys as they **sm**ə**℧k**_**Verb**_ into the cold air was breathtaking.
[smə**℧**k] → the gas-based substance emerging through chimneys

(7) They **sm**ə**℧k**_**Verb**_ the cigar.
[smə**℧**k] → the drawing in and exhaling of the gas-based substance by people

(8) Mariko went out for a **sm**ə**℧k**_**Noun**_
[smə**℧**k] → the drawing in and exhaling the gas-based substance by a person

(9) Lorena had to **sm**ə**℧k**_**Verb**_ the fish
[smə**℧**k] → the exposure of foodstuff to a gas-based substance for flavoring and/or preserving it.

(10) Sylvia decided to **sm**ə**℧k**_**Verb**_ the house
[smə**℧**k] → the exposure of an enclosure to a gas-based substance to rid it of pests.

(11) The **sm**ə**℧k**_**Noun**_-house had to be painted this summer
[smə**℧**k] → the property related to the gas-like substance or any aspect of its creation.

(12) Do you have a **sm**ə**℧k**_**Noun**_?
[smə**℧**k] → the instrument from which gas-based substance is drawn in and exhaled

**Figure 2:**
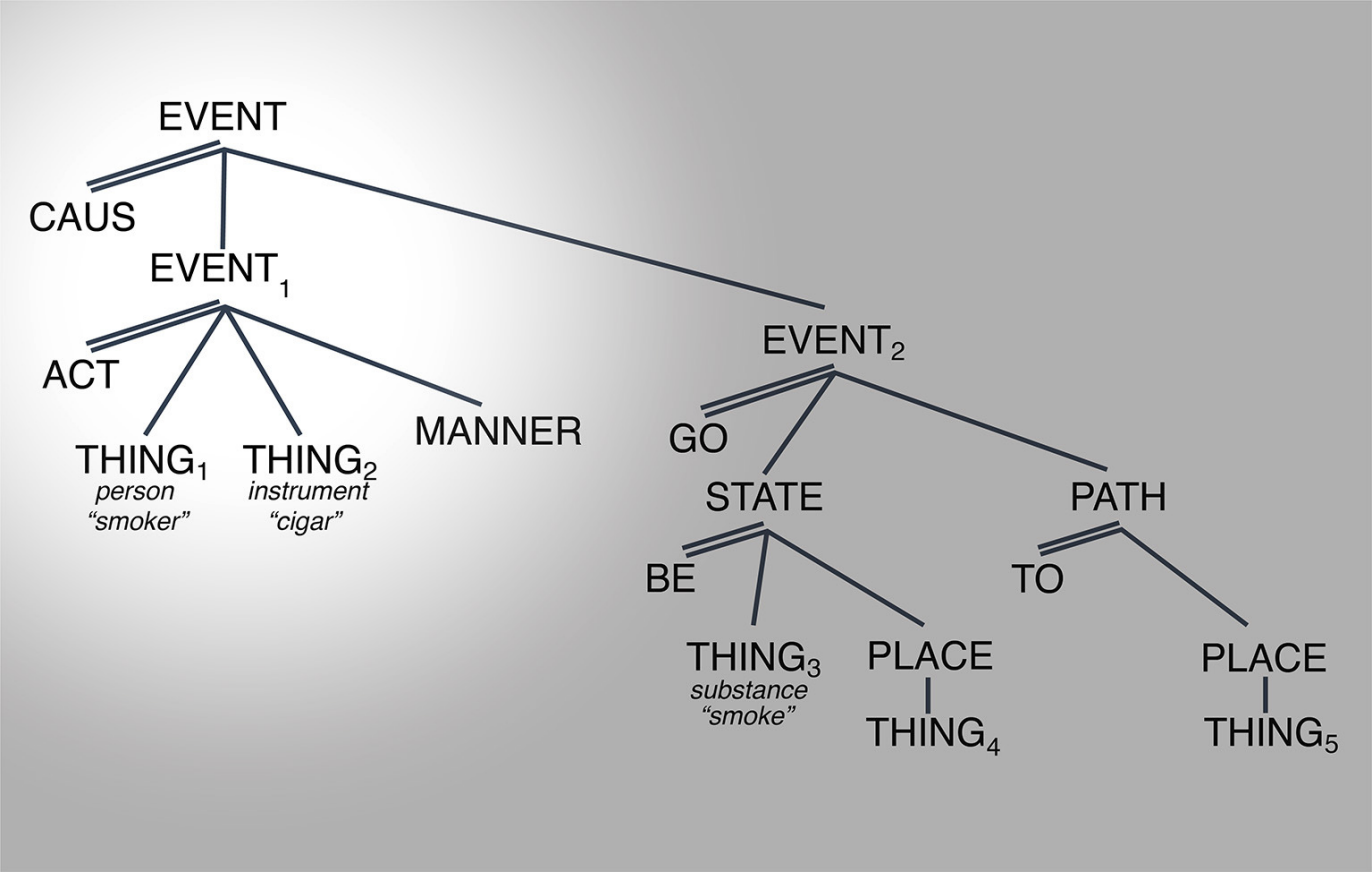
Conceptual representation for “They smoke a cigar”. Spotlight indicates salience.

**Figure 3:**
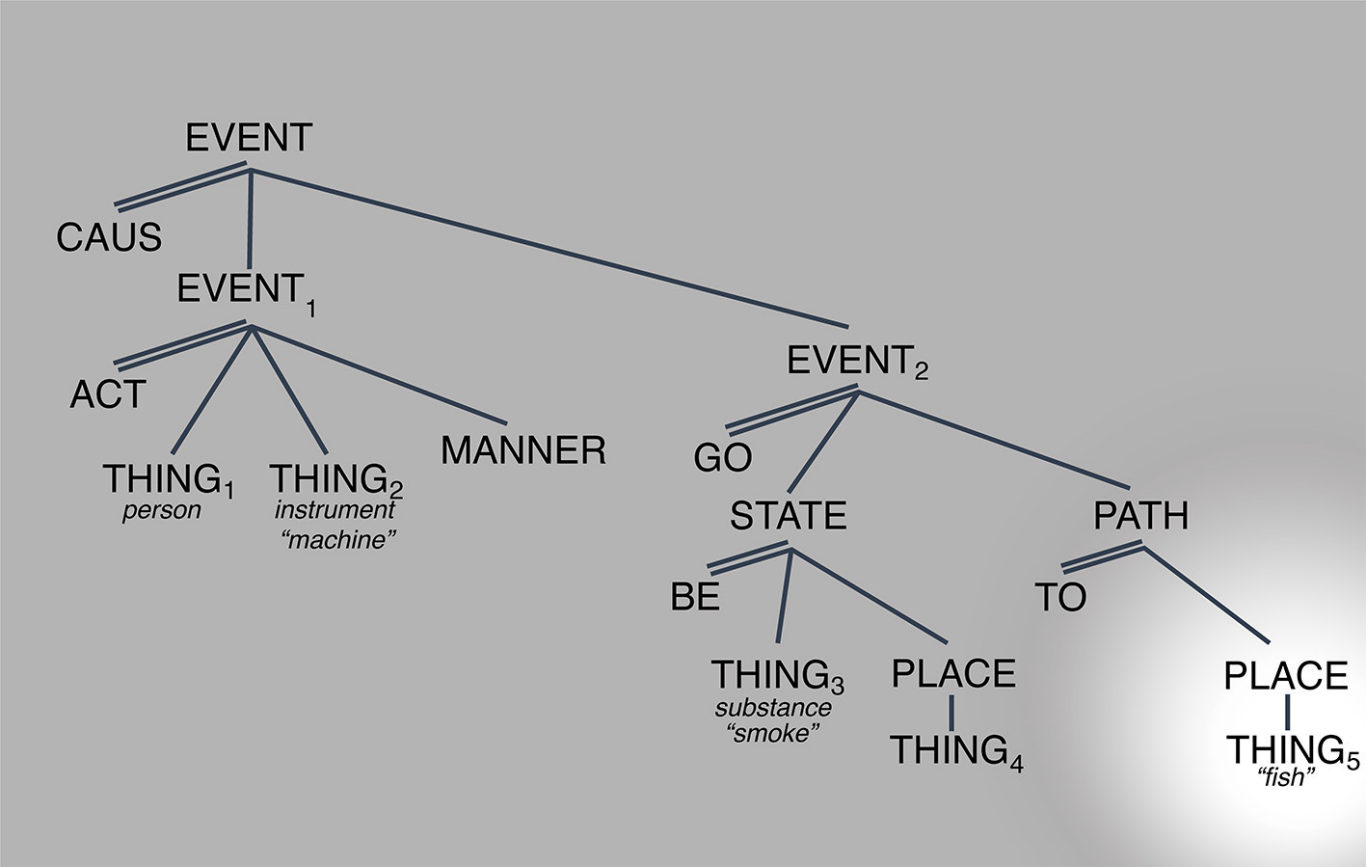
Conceptual representation for “They smoke a fish.” Spotlight indicates salience.

The key observations from these examples are (i) that syntactic category does not predict meaning: the same part of speech can give rise to different meanings (e.g., 4 and 12) and different parts of speech can give rise to the same meaning (e.g., 4 and 5), (ii) that even though the various uses of the pronunciation [**sm**ə**℧k]** bring up different interpretations or readings, these interpretations are not unrelated from each other. Indeed, the interpretations can be seen as *dependent* on each other, such that none can exist without the other.^7^ Figures 2, 3 below provide possible representations of this observation.

Figure 2 shows a representation of a situation of SMOKING^8^ as in (8) “They smoke a cigar” which involves an event with two (sub)events, an ACT-event (EVENT_1_ with two arguments, THING_1_ (person) acting on THING_2_ (cigar) in a specific MANNER) and a GO-event, where the consequence is played out such that THING_3_ (gas-like substance) emanates from THING_4_ (coreferent with THING_1_) onto THING_5_ (location of EVENT_1_).^9^

The first observation is that this structure contains the minimum number of elements necessary to capture our understanding of that sense of smoking. The second observation is that for each segment of the structure to be interpretable, the rest of the structure has to be activated as well. The argument THING_1_ represents the “smoker” only because it is the first argument of the ACT-event in which the second argument is a “cigar.” The ACT-event (EVENT_1_) takes place in a specific MANNER and gives rise to a specific consequence. So, the interpretation of any one referent as a participant in a specific situation is contingent on the rest of the referents associated with the conceptual structure that organizes that situation. There is no core vs. periphery to the structure.

The third observation is that readings that appear unrelated can be derived from the same structure simply through the manipulation of the referents associated with the arguments (THING_1_ through THING_5_). For example, the interpretation of (9) above “Lorena had to smoke the fish” or (10) “Sylvia decided to smoke the house” results from making the referent of THING_2_ “smoking machine” and THING_5_ to “fish” and “house,” respectively. This is shown for sentence (9) (modified) in Figure 3 above.

**Figure 4:**
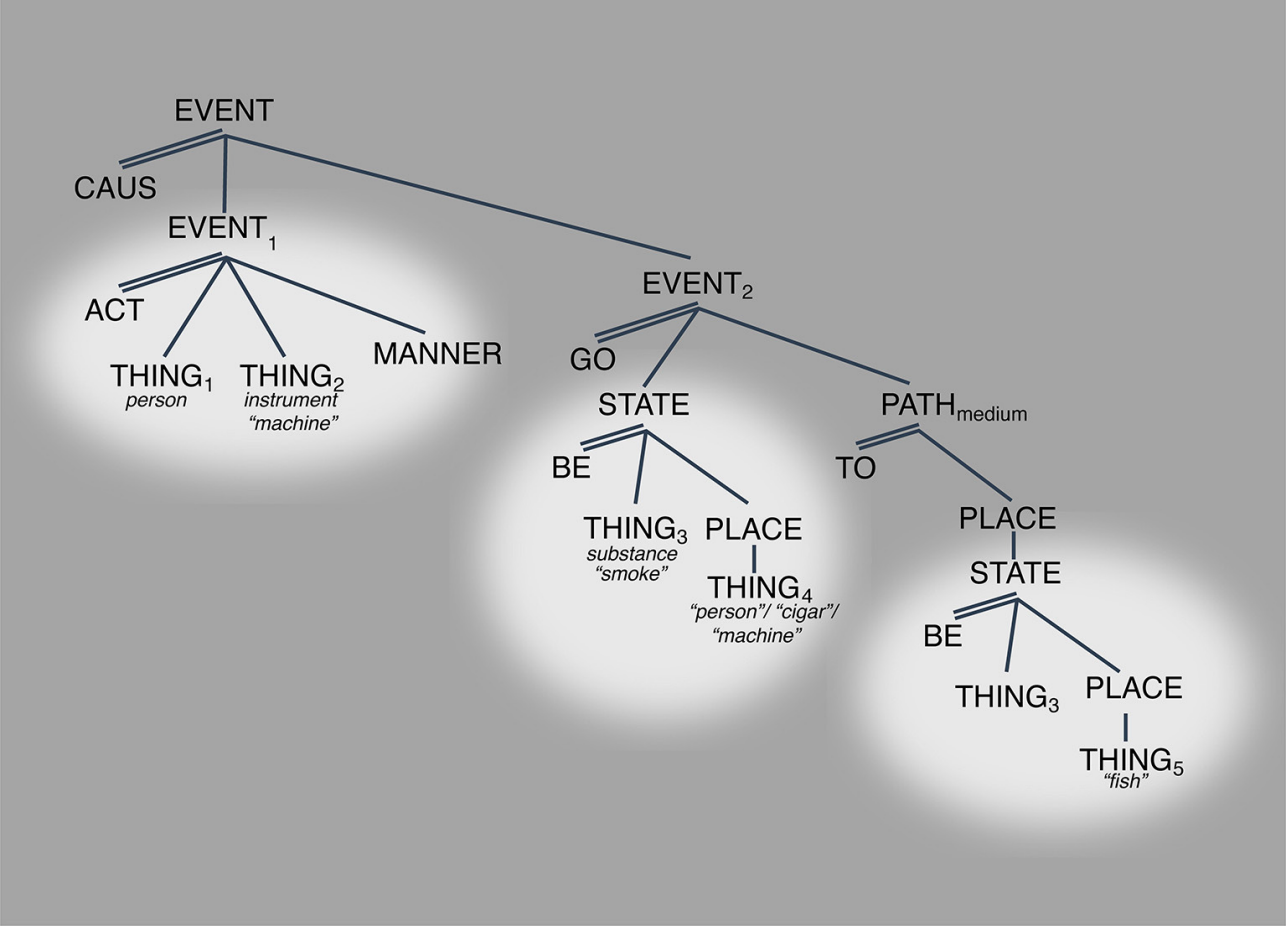
LCS OF SMOKE with many “meanings”. Spotlight indicates salience.

Relatedly, Figure 4 shows the set of readings that can be derived by manipulating the referent of THING_4_, the source of the smoke as in “The fire gave off a lot of smoke” or the path of the smoke: “The view of chimneys as they smoke into the cold air was breathtaking” [examples (5) and (6) above, respectively]. The case of “chimneys smoke” is interesting because it brings up the question of how much of the LCS, specifically the first causal subevent, must be active when this “path” reading is invoked. The proposal is that although backgrounded, the first subevent must be part of the activated LCS. The reason is that knowing what “chimney” means, knowing its LCS, means knowing that it serves as the path through which the gas-substance emanates, which is generated through subevent 1: the activity of an actor on some kind of combustible object in some manner (Figure 4).

The overarching point is two-fold: (i) that regardless of which part of the structure is salient the whole structure must be activated^10^ and (ii) that the various “senses” emerge simply when arguments that normally are left underspecified are specified. The claim is that this contrast in specification alone is enough to make salient a part of the structure that normally remains non-salient, in the background.^11^

At this point, it is possible to argue that the many meanings of [smə**℧**k] illustrate not a lack of boundaries in meaning, but rather an “expansion” of the categorial boundaries of a set of otherwise discrete representations. This would maintain the hypothesis that word meanings are discrete entities. But such a possibility is not tenable. Here are two reasons: (i) assuming a categorial separation would make the vast similarities across all the uses just described a coincidence, the least conservative position, and (ii) not only are the uses unifiable into one conceptual representation, SMOKE, but this conceptual representation is itself unifiable into a *class* of conceptual representations with which it shares algebraic properties. That is, none of the components in SMOKE is disconnected from other “concepts” with which it shares structure. For example, the ACT^12^ function (EVENT_1_) is connected to other activities that lead to ingestion (e.g., “eat,” “drink,” “inhale,” “absorb,” and “take in”), and these together connect to an even larger class of concepts that convey causality in more linguistically explicit ways, e.g., “break,” “kill,” “push,” and so on. The GO function is connected to other situations of transfer, e.g., “travel,” “move,” “send,” “give,” and “walk/run.”

The connections continue. To know the meaning [smə**℧**k] is to have some conceptual representation for FIRE which itself connects to one of HEAT and BURNING and the properties of the fuel being released within the gas-like substance during combustion, regardless of whether a pronunciation for it exists in the language of the individual.^13^,^14^ Furthermore, those conceptual representations themselves connect with others, in an ever-expanding “conceptual fabric.” To be sure, we do not expect that such a conceptual fabric would be present in the same way across all members of a given speech community. And that is because the degree to which knowledge of the world is encoded with any level of specification, such knowledge is highly dependent on the life experience of the individual, and the individual's cognitive make up, including their context-sensitivity (e.g., Zhang et al., 2022); factors that are likely to vary, and sometimes quite significantly, across individuals. The point remains though that the seamlessness with which conceptual structures must support each other during language comprehension is not consistent with the existence of an independently motivated categorial system. Instead, the experience of categoricality, of discreteness, if it exists at all, is epiphenomenal. It emerges from the salience created by variation in argument specification, and plausibility expectations induced by the utterance into which the lexical item is composed.^15^

#### 1.1.2. Challenge 2: How to account for word meaning boundaries when the meaning cannot be totally segmented: the case of English [hæv]

English *have* presents us with the opportunity to investigate a multiplicity of meanings when not only the pronunciation but also the syntactic configuration remains the same. Previous work has already shown how the many meanings connecting coincidental location and possession can be successfully unified into a conceptual representation (e.g., Zhang et al., 2018, 2022; Zhang, 2021).

Consider the meaning of [hæv**]** “have” in the following sentences:

(13) The tree has a car (next to it) → coincidental location
(14) The little girl has a car (in her hands) → non-coincidental location
(15) The woman has a car → alienable possession

a. …that she stole
b. …that she borrowed
c. …that she rented
d. …that she subscribed to (a zip-car)
e. …that she leased
f. …that she bought

(16) The car has bumpers → inalienable possession
(17) The car has a weak engine → inalienable possession.

The above examples and classification evidence the following: that the meaning of [hæv] spans a constrained set of interpretive possibilities from location through alienable possession and inalienable possession (e.g., Koch, 2012; Zhang, 2021). Moreover, whereas each instantiation occupies a specific point in the space, the space itself appears continuous. For example, cases (19.a) through (19.f) differ from each other in terms of legal responsibility from one argument to the other across each of the cases, but without a break from each other. Moreover, as the legal responsibility between arguments increases, thus making the relationship between them more inextricable, the degree to which one participant “the woman” is expected to exert control over “the car” also increases (e.g., one can trade with objects that one “owns” more than those that one has “rented” or “borrowed”). Crucially, these differences are established based on societal legal conventionalizations, there is no conceptual necessity that the specific cases exist, and no expected categorial system from which they should emerge. Those cases appear instead as possibilities that are afforded, precisely by the absence of a categorial break between them. This, in turn, enables infinite minimally differing conceptual realizations only limited by the practicality or usefulness of the distinctions, e.g., the possibility of making a distinction between “having a car” by borrowing from a friend vs. borrowing from a close family member or “having a car” based on a 3-year lease vs. a 5-year lease vs. a 10-year lease etc… the linguistic expression is the same, but the conceptual interpretation can be potentially infinitely ambiguous. The only constraint imposed by the system is that they be conceptualizable as lying within the “borrow” < –>“own” continuous space; itself underpinned by specified control-asymmetry/connectedness coordinates.

### 1.2. Interim summary

The two cases have made two points: conceptual associations to pronunciations of “word meanings” are (1) interdependent and (2) cannot be segmented. This directly questions the idea of “word meanings” as discrete entities listed in a repository for linguistic use, a mental lexicon. Instead, what the two cases above suggest is the necessity of a conceptual space that can be linked to linguistic materials (morphology, phonetics-phonology, and syntax) and that can allow for the creation of salience, all within a continuous space. The presentation of that model is the focus of the section directly below.

## 2. A model for meaning organization

Here, I present the minimal cognitive infrastructure that must be in place to support real-time linguistic meaning storage and composition. This model comes packaged as the Multidimensional Space (MdS) first discussed in Piñango (2019), and subsequently by Lai and Piñango (2019), and more recently by Zhang (2021), to capture patterns of linguistic phenomena previously thought to have clearly defined semantic role distributions, particularly in relation to agency, but which showed “fluidity” in how such agency was instantiated, thus questioning its categoricality. These cases involved linguistic constructions containing lexical and logical metonymy and location-possession relations (see Zhang, 2021, for a comprehensive summary).

Along the way, it has become apparent that the properties of the space support a broader and deeper understanding: as the space where *all* meaning associated with linguistic pronunciations is organized and stored. That is the hypothesis that I explore here.

The MdS is composed of the following: (1) a memory system that supports both storage (long term) and composition processes; (2) a system of “meaning” units that connects perceptual composites, “percepts,” which are potentially decomposable into generalizable conceptual structures;^16^ and (3) a mechanism that packages those conceptual units into the meaning categories that have direct morphophonological and syntactic reflexes (e.g., situations and participant roles within those situations). I explain each in turn.

### 2.1. Semantic and episodic memory in conceptual identification and storage

The MdS capitalizes on two fundamental and complementary memory spaces: semantic and episodic memory (e.g., Tulving, 1983, 2002; Baddeley et al., 2014: chapters 6 and 7). Semantic memory involves the accumulated experience of the world by an individual abstracted away from reference to any specific instance (Binder and Desai, 2011, p. 527). Episodic memory complements semantic memory in that it holds a memory for specific experiences; that is experiences that are time- and space-stamped (Binder and Desai, 2011, p. 527). The crucial distinction between the two spaces is thus conceptual specificity—whereas semantic memory is the space for abstractions and, to some extent, under-contextualized content, episodic memory is the space for the particular, experiential, and “autobiographical” content. Both memory systems hold the same conceptual substance indicating in turn that they are both contained within a shared memory space. Semantic and episodic memories are thus two poles within a continuum modulated by degrees of encoded direct experience. This is the domain that the Multidimensional Space articulates, the space that realizes all meaning, including linguistic meaning.

On the MdS, both subsystems share the *episode*; this memory unit finds a counterpart in the linguistic *situation*, a supramodal conceptual representation of a state or event, that specifies not only the participant roles and their relations but also their temporal and spatial referential properties. Situations are organized algebraically and are decomposable in terms of innate cognitive primitives.^17^ This internal articulation represents the unit for the identification, storage, and composition of situations in the world. Within the MdS the units are then *situation-episodes*. As the number of contexts increases in which a given *situation-episode* appears, the more general and therefore the more “semantic” the episode will be. The richer the episode is stored, with specifics of the context under which it was acquired, the more “episodic” it will be. This makes the distinction between episodic memory and semantic memory one not of structure but of perspective, modulated by how much experience-specific context is encoded with the situation-episode.

Situation-episodes are thus memory structures that package interactions between entities and between an entity and its environment. Episodic memory is the input mechanism to semantic memory while semantic memory includes situation-episodes that are abstracted from experience and generalized as decontextualized situations, that is, situation schemas. Situation-episodes provide structure to both specific (time/place stamped) episodes and generalized episodes that emerge from repeated experience. On this view then, episodic and semantic memory are two sides of the same domain. That is, although contextually richer, fully specified situation-episodes are never disconnected from the conceptual generalization, the generalized situation-episode, that they served to create in the first place.

Episodic and semantic memory systems connect as follows: situations enter semantic memory through episodic memory as contextualized situation-episodes. As the same situation-episode is identified in an ever-increasing number of contexts, they become increasingly decontextualized and therefore generalizable and possibly more accessible to lexicalization. That is, generalizability and lexicalizability are outcomes of the increased frequency of an individual's experience with the same semantic relations in an ever-increasing variety of contexts. The increased diversity of contexts is what allows the memory system to extract, as it were, properties of the situation-episode that remain constant. This makes them fundamental and generalizable. Properties that change across contexts are deemed less fundamental, and therefore less likely to be included in the generalized version of the given situation-episode, rendering it decontextualized. It is this possibility to “prune” the situation-episode of incidental properties what makes it lexicalizable. Being decontextualized, the situation-episode can be used in a greater variety of contexts. Decontextualization enhances conceptual combinatorial potential. On this view then, semantic and episodic memory together serve to organize concepts into structures to which language composition is sensitive.

Storage of specific/unique and generalized situation-episodes is the space from which linkages to pronunciation, i.e., lexicalization, take place. This is a space for linguistic meaning construal: the identification and composition of situation-episodes as the linguistic communicative process unfolds.

Finally, storage is principled: situation-episodes are organized as a parametrically constrained plausibility distribution.^18^ The parameters are connectedness and control-asymmetry. Points in the distribution approximate readings or senses. It is out of this parameterized space that situation-episodes are encoded and retrieved, and novel ones are composed. I describe each parameter directly below.

#### 2.1.1. Control-asymmetry

Control-asymmetry allows the processor to assess the degree of relative control power among the participants in a situation. In a two-participant situation-episode, an evaluation of high control asymmetry means that one participant is construed as having control over the other participant (and not the other way around). An evaluation of low control-asymmetry means that either participant can exert equal control over the other. Control asymmetry thus underpins perception of causality and causal chains, a fundamental property of human cognition (e.g., Talmy, 1988, 2000; Carey, 2009), and force transmission (e.g., Croft, 2012).^19^ Crucially, force transmission is by nature an asymmetric relation: the argument with greater control appears causally responsible for the relationship in which it appears (see Pinker, 1989; Croft, 2012 for further elaboration).^20^ Control asymmetry also interacts with animacy in interesting ways. The aspectual-verb sentence “The printer started my paper” gives rise to an agentive reading yet it has an inanimate-denoting subject. By contrast, the sentence “The little girl began the queue in front of the candy shop” yields a constitutive reading yet its subject-denotation is animate. The agentive vs. constitutive reading thus appears orthogonal to animacy considerations yet it is naturally predicted by control asymmetry distinctions—high control-asymmetry—between “printer” and “paper”—resulting in an agentive reading and low control-asymmetry—between “girl” and “candy shop”) constitutive readings (see Lai and Piñango, 2019 and Lai et al., 2023, for further elaboration, and Piñango, 2019, for connections to metonymy composition).^21^

#### 2.1.2. Connectedness

Connectedness refers to the degree of perceived (or hypothesized) spatio-functional relation between two participants. It ranges from incidental proximity, which is coincidental, to contextualized location (less coincidental), to proximity by functional connectedness, normally containment, to complete functional and spatial dependence, reflecting a systemic, non-coincidental parthood relation understanding. This progression implies that as the perceived connectedness relation between individuals moves rightwards, toward greater connectedness, the perception of the relationship becomes less coincidental and the individuals involved are interpreted instead as increasingly functionally dependent on each other.

The progression implied by incremental connectedness supports distinct visuospatial configurations: low-connectedness supports adjacency, mid-connectedness supports containment, and high-connectedness supports parthood configuration. This correlation suggests in turn a potential source for perceptual grounding of otherwise abstract cognitive inferences.

### 2.2. Control asymmetry-connectedness interaction: the model

**Figure 5:**
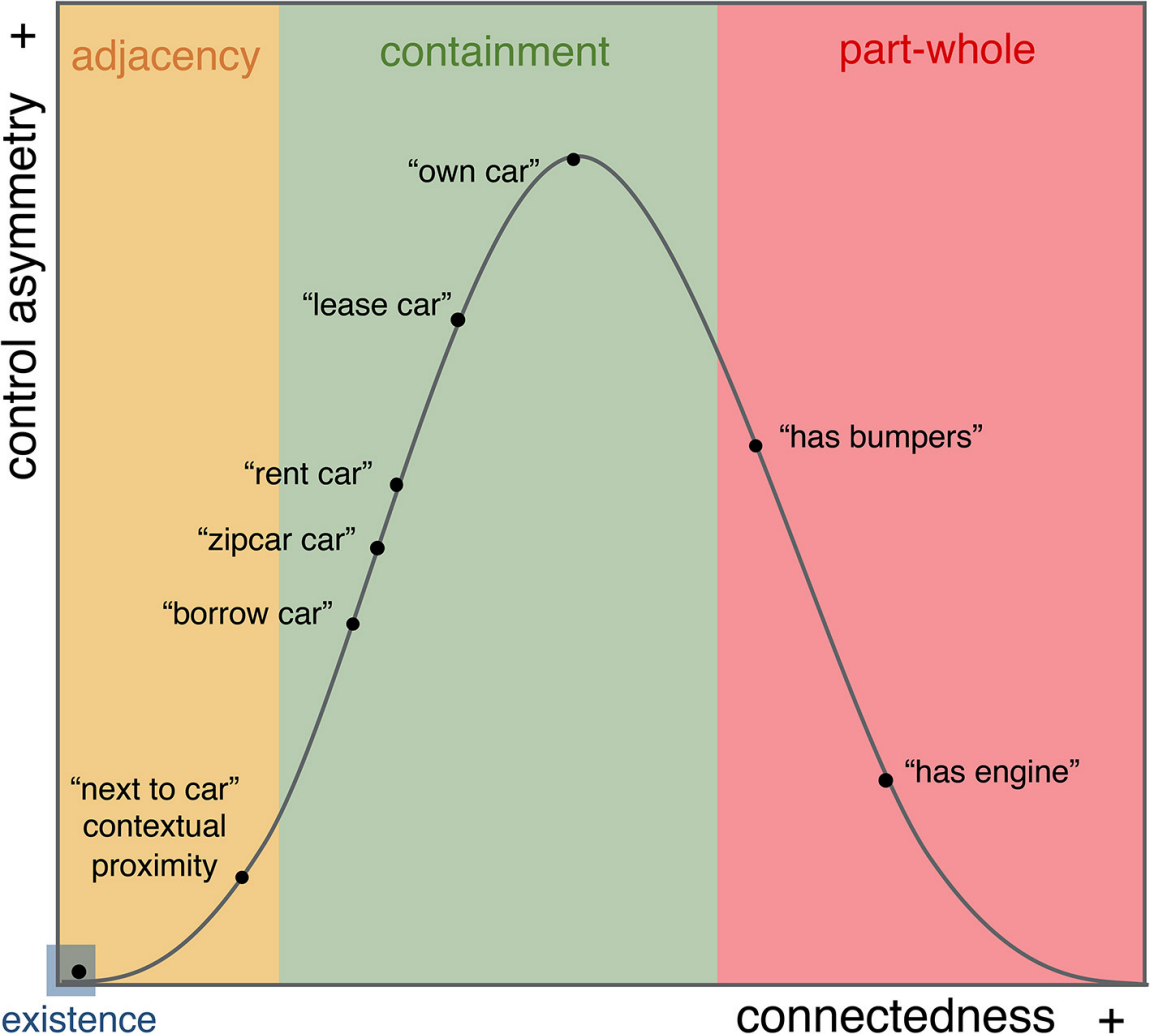
The Multidimensional Space with the location-possession distribution.

The conceptual distinctions afforded by *connectedness* can be integrated with differences in control hierarchy afforded by *control-asymmetry*. Specifically, low control-asymmetry is expected in situations of adjacency (e.g., coincidental location) and part-whole configurations (e.g., inalienable possession),^22^ the two extremes of the connectedness continuum, whereas high control-asymmetry is expected in containment configurations (e.g., cases of alienable possession). This is shown in Figure 5.

Evidently, while the parameters do not *create* situation-episodes, they guide their interpretation by imposing an evaluation of connectedness and control-asymmetry between the participants in the situation-episode. It is this imposition that allows us to naturally distinguish “car-renting” from “car-leasing” from “car-owning.” In these cases, the changes in control-asymmetry/connectedness stem from an evaluation of the extent to which each given participant is a controller or controllee with respect to the other. Those variations in evaluation are naturally encodable in the intervals that separate the three situation-episodes moving from less to more control-asymmetry/connectedness (Figure 5). From *connectedness*, we glean configurations of incremental physico-functional closeness, e.g., a car has a greater dependence on the controller in an owning situation than in a renting situation. From *control-asymmetry*, we glean causal hierarchies. This represents the basis for more semantic role relations which distinguish “owning” from “renting,” for example, “permission to use and trade x” vs. “permission to use x,” respectively. In both cases, there is control-asymmetry, but as the scope of permission increases so does the control-asymmetry, inducing the intuition that “owning” carries greater agency. In this way, these two constraints guide the processor in building a repertoire of situation-episodes in semantic memory, as it encounters them.

Discussion of the processor brings up the question of its involvement in meaning composition: whether it involves pointing to various locations in the conceptual space or whether it also involves the construction of situation-episode representations “on-the-fly.” The question so formulated speaks to the real-time constraints that the processor imposes on meaning construal, constraints that so far I have only addressed indirectly. A full discussion of the processing component of the model is outside the scope of the article. But here is the idea. One fundamental assumption of a lexically driven processing system involving a semantic memory space like the MdS is that during language comprehension lexico-conceptual elements of a situation-episode are brought together through morphophonological and morphosyntactic composition. Those compositional subsystems serve as the scaffolding for linguistic comprehension, the objective of which is the building of a contextually plausible situation-episode. Such a building process can take place either through direct identification of a previously stored situation episode that matches in all aspects the incoming one, or the identification of a partially matching one, whereby (a) the predicate and the participants are recognized (found a match for) but composition with the specific participants is new, or (b) some but not all components are recognized triggering the creation of a *new* situation-episode. In all cases, composition happens on-the-fly, within the space. The difference is how much pre-existing situation episodes at all levels of generality are able to support the building of the new one. So, the compositional demands are gradient, conditioned by the degree to which the processor can find matches in the space to the input situation-episode.

Interestingly, these various matching possibilities can be linked to well-known processing patterns by modeling the recognition process as an evaluation of plausibility: the closer the match between an incoming situation-episode and one stored in the MdS, the more plausible (and faster) the expression will be evaluated and therefore comprehended; and the more “novel' the situation, the less plausible it is judged, and the greater and more delayed the cost of interpretation. This is what processing observations of the past 50 years show.

Another property of the continuous space is that it does not distinguish functional from biological factors: both are part and parcel of the knowledge system that allows us to parse the world. This has the added value of blurring the distinction between traditional contrasts such as abstract vs. concrete, animate vs. inanimate, and natural vs. manufactured, which, while intuitive, have notoriously resisted independent formal motivation. Within the MdS, the properties may exist but not necessarily as organizing principles of the space.^23^

I conclude this section by listing several payoffs of the model: (a) situation-episodes, i.e., algebraic configurations, are “mini-stories”; capturing ways in which we understand the interactions between elements around us. (b) situation-episodes exist within a continuous space that prevents structural discreteness and allows for generalizability across conceptual dimensions (e.g., temporal and informational) giving rise to so-called “metaphorical” senses.^24^ These relations are not primitives of the space, they are *generalizations* over multiple situations that share specific control-asymmetry-connectedness calibrations, e.g., low control-asymmetry with low connectedness leading to coincidental proximity and low control-asymmetry with high connectedness to part-whole arrangement. Consequently, (c) the boundaries between situation-episodes and any of its components are not categorical but result from biases within the space created by continuous exposure to specific *kinds* of situation-episodes.

## 3. Discussion: back to SMOKE and HAVE

**Figure 6:**
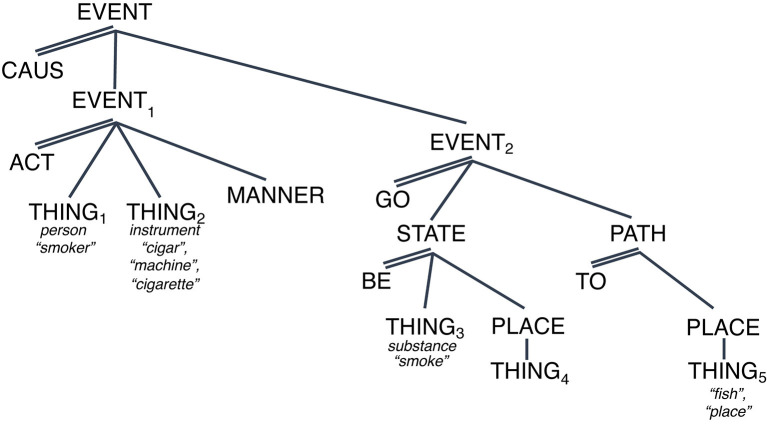
LCS of *SMOKE*.

So, what are the implications of the model for the understanding of the meaning of “smoke” and “have”? How do we reconcile the algebraic representations with the continuous space? The answers to these questions can now be stated straightforwardly. Figure 6 shows the LCS for SMOKE which is stated in terms of a causal relation between two events: EVENT_1_ involves the actor and instrument of smoke, and the other, EVENT_2_ involves the transfer of gas from the instrument to elsewhere (Figure 6).

**Figure 7:**
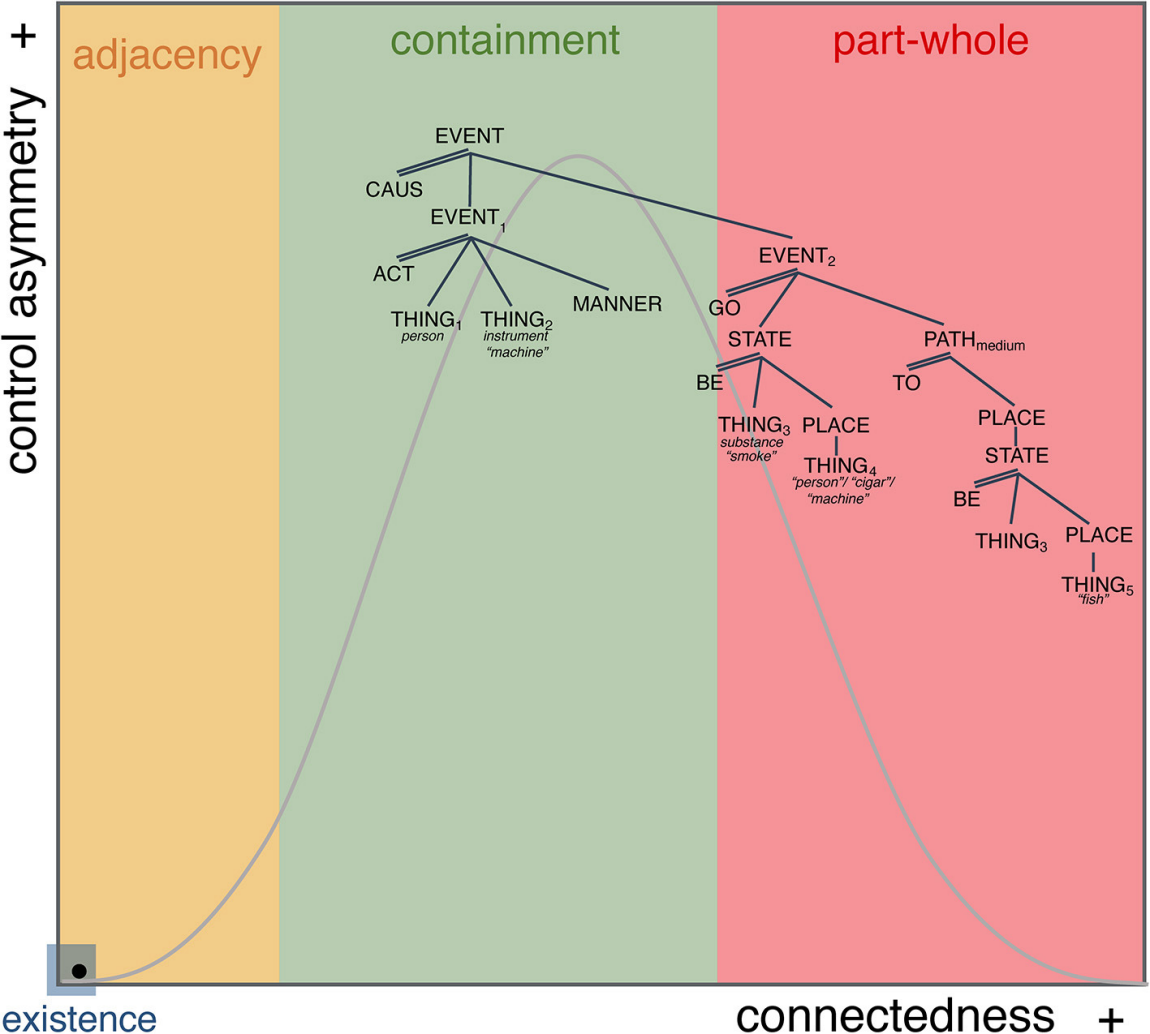
LCS of *SMOKE* within the MdS.

Figure 7 illustrates how the LCS “lives” within the continuous space. What we observe for SMOKE is a causal structure that is distributed across the MdS such that EVENT_1_, the ACT-event, is understood within the high control-asymmetry/mid-connectedness (containment) space and EVENT_2_, the GO event, is understood within the low-control asymmetry/high-connectedness (part-whole) space. High control-asymmetry space indicates the interpretation that in its ACT, THING_1_ has absolute control over THING_2_. Being in mid-connectedness space signals that there is a functional relation between those two arguments but that it is not part-whole, e.g., the relation between a person and a cigarette, or a person and a smoke machine. Event_2_ is in low-control asymmetry space because in the relation relation between THING_3_ (*locatum*, e.g., gas), THING_4_ (source, e.g., person-cigar and smoking machine), and THING_5_ (goal, e.g., air and fish) none of them have greater control over the other, they are not understood in terms of a hierarchy, at the same time, they show interdependence through containment which places their relation in high-connectedness space, e.g., person-cigar and machine contain and emit the gas, and air and fish contain gas once emitted (Figure 7).

**Figure 8:**
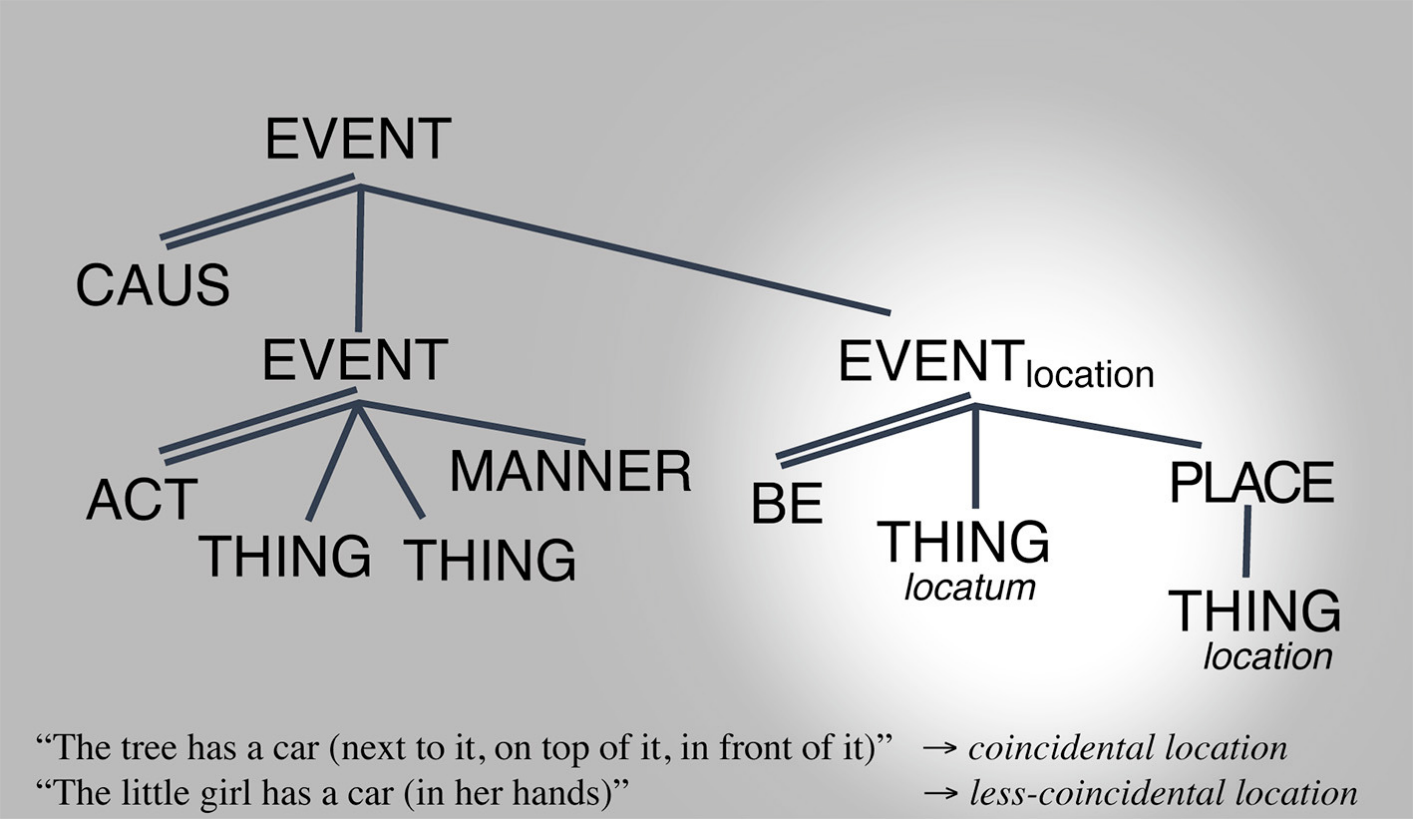
Location space reading. Salience indicated by the spotlight.

**Figure 9:**
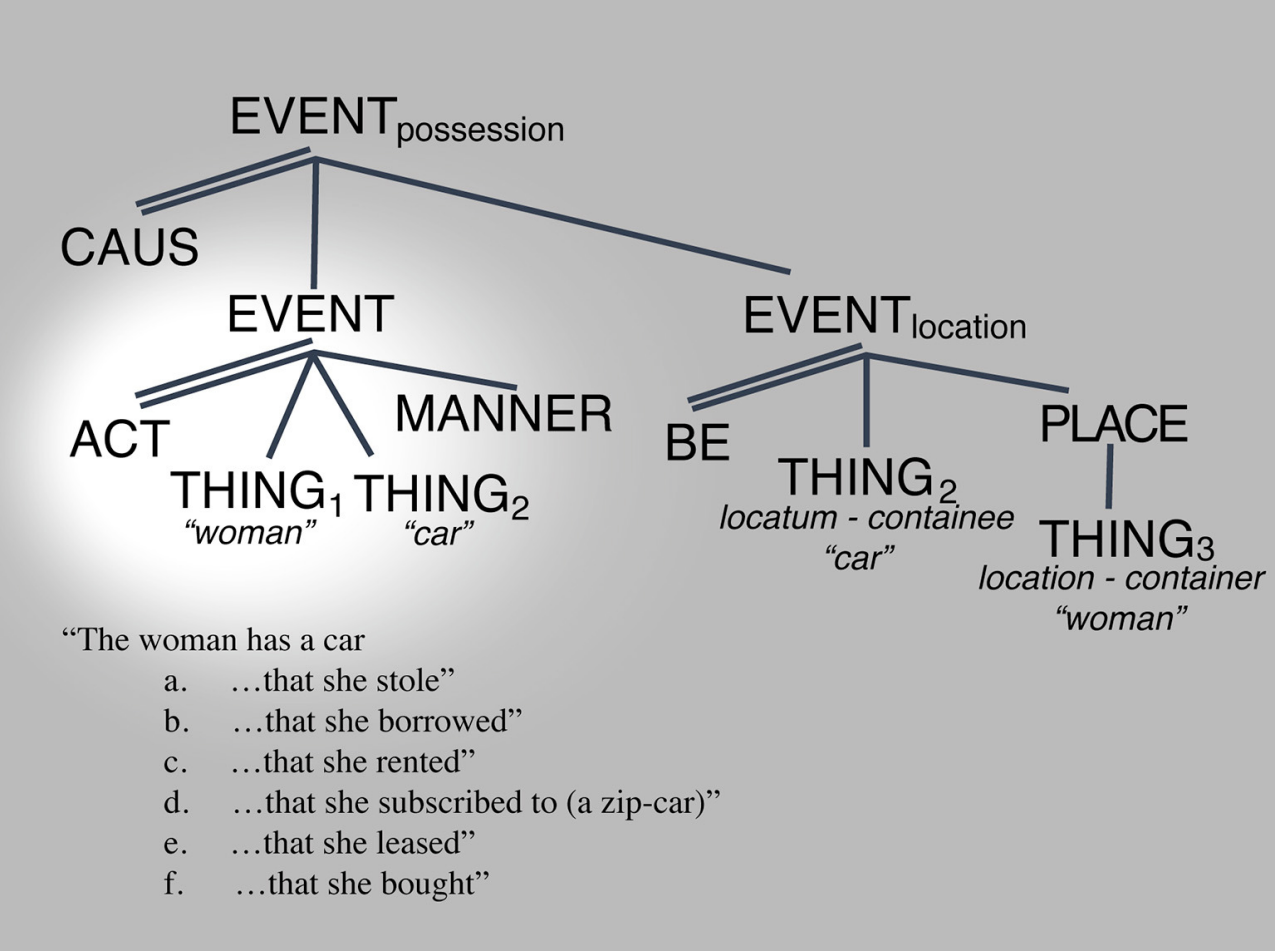
Alienable possession space reading. Salience indicated by the spotlight.

**Figure 10:**
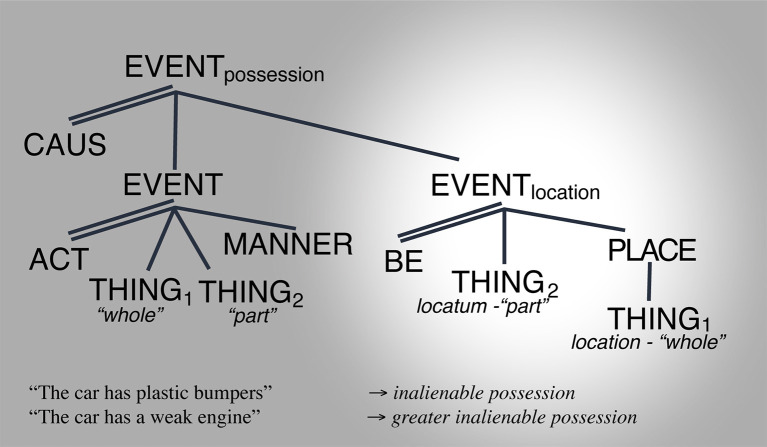
Inalienable possession space reading. Salience indicated by the spotlight.

Turning to HAVE, Figure 8 through Figure 10 illustrate the various ways in which the LCS of *have* can show its readings through changes in salience. (25) shows the locative reading, (26) shows the alienable possession reading, and (27) shows the inalienable possession reading (Figures 8–10).

**Figure 11:**
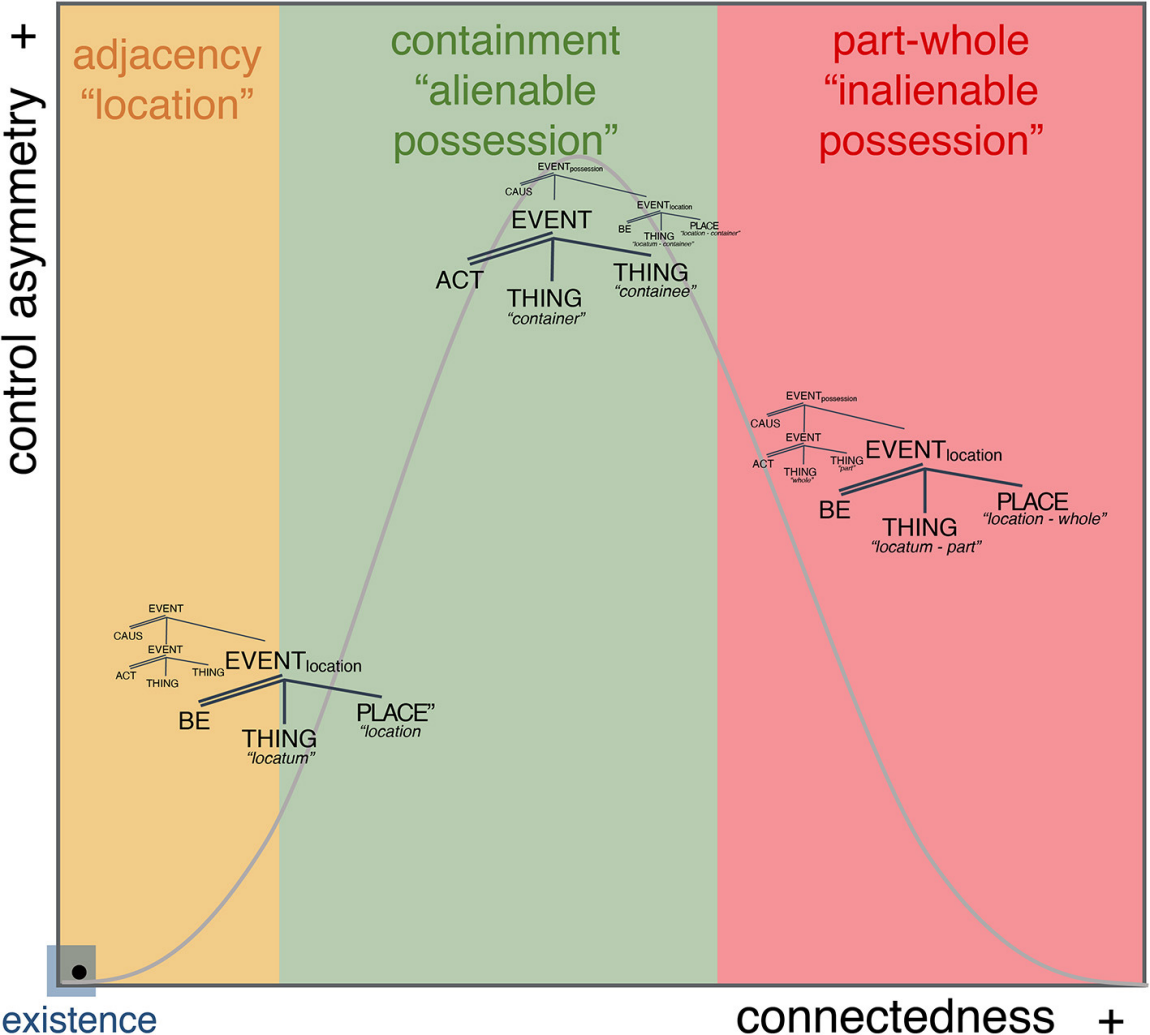
*HAVE*'s LCS within the MdS.

In this way, HAVEs' conceptual configuration is found throughout the continuum connecting an infinite number of readings from locative with infinite degrees of incidentality to alienable possession—with infinite degrees of mid-connectedness and mid- to high-control asymmetry—to inalienable possession with infinite degrees of understanding of functional part-whole integration. This is illustrated in Figure 11.

In summary, here I have explored two challenges to meaning discreteness and discussed in some detail two cases that illustrate them. These two challenges present us with the situation that our intuitions about word meaning discreteness appear at odds with how pronunciation-meaning associations behave whenever they are outside of a sentential or phrasal context: impossible to circumscribe. The behavior argues for a conceptual system that is based on a continuous space that allows for the encoding and composition of conceptual relations between participants. That is the role of the Multidimensional Space, a continuous memory-based system where conceptual units, situation-episodes, are encoded and further composed constrained by control-asymmetry and connectedness parameters.

The variability in semantic behavior of two cases, English [smə**℧**k] and English [hæv**]**, reveals that the lexico-conceptual representations that codify their respective meaning variability naturally manifest the differences in terms of differences in control-asymmetry–connectedness interactions. In this way, we can see how the specific relations and participants that the LCSs organize are systematically supported by a continuous space to evaluate, identify, and store situation-episodes, the units that codify our life experience, our “parsing of the world.”

## 4. Conclusion

In the absence of structural discreteness, how should we understand word meanings? Answer: we should understand them as conceptual structures associated with a pronunciation made salient by the interaction with other conceptual structures in the context of the linguistic expression. What gives rise to the experience of discreteness? Answer: The sense of salience that context provides supported by the episodic nature of sentence comprehension. How can we allow for linguistic meaning *composition* as based on word meaning interdependence and unboundedness? Answer: What language composition provides is a kind of “coordinate set” to a meaning space within the MdS. This is a rather different way of thinking about meaning composition. The results from the Multidimensional Space presented here are one step in thinking in that direction.

An MdS approach to the linguistic meaning structure has implications for other fundamental components of language use which are outside the scope of this article. They involve (i) the role of frequency which has long been recognized as having a significant impact on the facilitation/inhibition of language comprehension processes. Within this framing, not only the frequency of exposure but also the diversity of exposure by an individual, matter to the structure of the control asymmetry–connectedness interaction distribution that such an individual can generate. This is connected to (ii) the role of linguistic patterns. Different languages make salient different patterns. The greater the frequency with which a conceptual configuration is linguistically conveyed, the greater the salience associated with that situation-episode *vis-a-vis* another situation-episode that is afforded by conceptual structure yet is not readily conveyable in that language, e.g., think Spanish “grima” or Portuguese “cafuné,” words notorious for having only indirect word translation in English.^25^ These differences in linguistic exposure by individuals could represent the basis for increased/reduced linguistic innovation. That is, the MdS could be the space for the creation of novel paths in the conceptual structure; paths that guide innovations in language use and ultimately change.

Finally, continuous space for word meaning realization has implications for (iii) the role of attention as a linguistic-meaning processing mechanism. A continuous space for word meaning realization means that all linguistic meaning composition is *of a kind* and that there are no “standard” vs. “non-standard” ways to produce linguistic meaning. This implicates a whole family of compositional processes such as idiomatic expressions, metonymy, and metaphor connected under the “figurative” language umbrella; and normally considered “peripheral” (e.g., Jackendoff, 1997; Piñango et al., 2017). Under an MdS-based view, a *sense* of a word is the placing of attention in a specific node in conceptual structure led to it through linguistic structure. This means, for example, that when the word “ocean” is used in the phrase “ocean of grass,” it is simply leveraging the fact that, conceptually, pronunciation is fundamentally referring to a vast container (mid-control-asymmetry and mid-connectedness). The fact that its content is typically understood to be salty water is not inherent to the LCS, it is just another variable that the language allows us to access and therefore manipulate, and in doing so, allows us to further expand our repertoire of situation-episodes.

## Author contributions

The author confirms being the sole contributor of this work and has approved it for publication.

## Footnotes

^1^Throughout this article, I use the formalisms of conceptual semantics (e.g., Jackendoff, 1983, 1990, 1997, 2019; Pinker, 1989; Levin and Pinker, 1991) to represent the possible conceptual structures that our mind stores, and composes with all its dynamics, including language comprehension. Following convention, I use all caps to denote the concept.

^2^Throughout, the term pronunciation refers to pronunciation of lexical units. When relevant, I use the phonetic (IPA) representation for the English pronunciations to maintain the awareness that the link between language and the conceptual system is pronunciation in oral or visual form, not script. Finally, following convention, I use all caps to denote concepts.

^3^Although relevant, discussion of compositionality is beyond the scope of this article. See, however, Werning et al. (2012), for a whole volume of extensive exploration of this principle and specially Baggio et al.'s chapter on the processing consequences of compositionality, which delves deeply into the psycholinguistic implications of the principle (Baggio et al., 2012).

^4^Traditionally, “Idioms” or “Idiomatic expressions” are discussed in the literature precisely because at face value, they violate the most basic assumptions about compositionality yet they are widely observed across languages of the world. Moreover, they appear to be part and parcel of the same linguistic system that gives rise to non-idiomatic counterparts, and therefore their behavior is potentially informative to models of language (see Fraser, 1970, for early discussion in the context of generative grammar, Jackendoff, 1997, for a general view of the issues and a specific proposal of a model, and O'grady, 1998, for an exploration of the syntactic properties of idiomatic expressions that link them to mainstream syntactic theorizing).

^5^Here is a passage from Quinn's “My Ishmael” (1998, p. 38–39) that neatly illustrates how predictability is achieved only through context: “The word *culture* is like a chameleon, Julie. It has no color of its own but rather takes color from its setting. It means one thing when you talk about the culture of chimpanzees, another when you talk about the culture of General Motors. It's valid to say that there are only two fundamentally different human cultures. It is also valid to say that there are thousands of human cultures. Instead of trying to explain what *culture* means when it's all by itself (which is almost impossible), I'm just going to explain what *I* mean when I say “your culture.” All right?”.

^6^See also Bierwisch and Schreuder (1992) for same examples of this same pattern.

^7^Note that the diversity of meanings can vary with syntactic use but is dissociated from it. Consider the minimal pair: “smoking heavily” vs. “heavy smoking,” where the first use is as a verb as evidenced by the adverbial modification, and the second, a noun, as evidenced by the adjective modification and yet in both cases, the same situation is invoked.

^8^Again, following convention, I use all caps to denote the concept, the construct that LCSs are hypothesized to capture.

^9^A reviewer asks whether THING_5_ must be generated in the case of (8) since the location of the smoke after being exhaled by the smoker and cigar need not be made explicit. The answer is that understanding of (8) means that not only is there smoke being generated but also it is going *somewhere*, by default, the local environment where smoking is taking place. This is the understanding that an underspecified THING_5_ supports, and the reason it must be generated in the LCS associated with the sentence.

^10^A reviewer asks whether the meaning composition would happen differently in languages with richer derivational morphology than English, allowing for certain components of the situation-episode not to be activated until the corresponding affix, say, introduce it. While the data are not conclusive, the evidence so far suggests that the role of derivational morphology is like that of syntax to induce sense-salience. The overarching observation is that the processor is predictive. In the context of meaning composition, predictability means that the processor will generate the most complete situation-episode it can at each point in the unfolding of a sentence even if the given situation-episode has not been made linguistically explicit. So, the semantics introduced by derivational morphology would have a confirmatory role. They would directly address attention to a specific part of the LCS that has been generated but was up to that point underspecified, in the background (see Ferreira and Chantavarin, 2019, for extensive discussion of predictivity in the context of traditional integration-based approaches to sentence comprehension).

^11^In the cases that I am considering here, salience is motivated through the specification of an argument in the LCS of the sentence. For example, in the sentence “They smoke a cigar,” THING_5_, the end-point for the gas-like substance is left unspecified. This allows the arguments of (sub)event_1_, < “they,” “cigar”> the ones made linguistically explicit, to be salient. In “They smoke a fish,” THING_5_, not THING_2_, is specified and this enables the shift in salience to PATH function in (sub)event_2_.

^12^The ACT function in its dyadic form involves canonically an ACTOR/agent and UNDERGOER/patient. As such it underpins all situations that involve dynamicity and control asymmetry, in addition the ACT-event serves as locus for a MANNER where the relation between agent and patient is specified (Pinker, 1989, p. 192–195).

^13^These observations are of course not new. They have been leveraged by all approaches to meaning that capitalize on a conceptual system to capture patterns of linguistic meaning including Conceptual Semantics, Frame Semantics, Prototype Theory, and of course, Conceptual Semantics. See Talmy (2019), and references therein for recent discussion of the shared and not shared aspects of lexico-conceptual approaches to meaning structure.

^14^The question of how the existence of a pronunciation matters for the quality of a concepts representation is in fact an empirical question. Evidence from Pyers and Senghas (2009) strongly suggests that it does, further suggesting that the role of language is not limited to conveying thought. It extends to providing further structure to thought, possibly in the form of differences in salience thus making some aspects of a concept more accessible to lexicalization and retrieval than others.

^15^See MacDonald (2013), for an in-depth exploration of sentence comprehension through the impact of frequency and plausibility in sentence production.

^16^A reviewer wonders about the necessity for percepts of any sort; why would the system not assume concepts right away? It is the case that the MdS assumes a set of conceptual primitives. Indeed, those are the ones that allow the decomposition of the percepts into a structure that the MdS can organize. So, conceptual structure is assumed from the get-go. The percept is merely the “raw” input over which conceptual structure is imposed. This input need not be linguistic, it can also be visual, haptic, olfactory and any other potential sensory source (e.g., Jackendoff, 1990, 1997, 2002).

^17^A natural question is why I cannot consider word meanings as the conceptual structures that articulate episodes. One reason is that these structures are not in a one-to-one correspondence with our intuition about word meanings. This is shown most clearly the discussion of [smə**℧**k] above, which demonstrates how the same conceptual representation can be associated with different interpretations, arguably “meanings,” of the same pronunciation. Another reason is that these structures or components of these structures are shared across multiple interpretations calling into question discreteness itself. A third reason is that conceptual structures are not finite. They appear finite only because when I discuss them, I do so in the context of its link to a pronunciation in a given context, a finite expression. A more fruitful way to think about conceptual structure and any “segment” within it is as “articulators” of my experience that can, but need not, be associated with pronunciation (e.g., Jackendoff, 1990, 2019; Bierwisch and Schreuder, 1992; Talmy, 2000, 2019).

^18^The existence of this space was originally motivated by well-known interactions between three distinct conceptual domains—existential, locative, and possessive—whose relevance has been shown through diachronic and synchronic linguistic modeling (e.g., Clark, 1978; Jackendoff, 1983, 1990), in language acquisition (e.g., Pinker, 1989), and in language typology and variation (e.g., Lyons, 1967; Koch, 2012). Our starting point is the shared insight that to the extent that they are visible through language, these domains may not be categorically distinct.

^19^Klein and Perdue (1992) argue convincingly for the existence of a basic variety which they describe in this way: “all our learners, irrespective of source and target language, develop a particular way of structuring the utterances which seems to represent a natural equilibrium between the various phrasal, semantic and pragmatic constraints.” A key element in their hypothesis is a constraint which they call the “controller principle”: the NP referent with highest control comes first (p. 48–49). This principle leverages the degree to which language users show a bias toward linguistic privileging a referent if it is perceived, expected, or intending to be in control of the other referents. The claim of the MdS is that control is visible to language through the evaluation of control *asymmetry*, a parameter that imposes a calculus of control between participants as they are brought together during language use by situation-episodes.

^20^In this manner, control-asymmetry subsumes the conceptual representation of agency. An agent is an actor that is perceived as having self-control and perceived as exerting it over another, the undergoer (Jackendoff, 1983, p. 181; Talmy, 1988; Pinker, 1989, p. 31; Croft, 1994; Carey, 2009, p. 217). Agency is also subject to degrees: the more controller potential a participant is construed to possess over another in a situation, the more likely they will be taken as an “agent”. This impacts causality which is normally understood to be initiated by an “agent”. However, more nuance is needed. The sentence “The heavy rain ruined the man's shoes” involves an (accidental) causal relation between the two participants (heavy rain and the man's shoes) yet does not encode agency as normally understood (Carey, 2009, p. 217). So, what is required is a system that allows us to distinguish causal events without grounding them on the sentience of the initiator, while at the same time allowing for sentience and animacy to be considered. That is precisely the role that the MdS naturally plays.

^21^Consistent with the use of control-asymmetry as a space for evaluation of situation-episodes, Saxe et al. (2005, 2007) report that preverbal infants compute agency from patterns of interaction among entities and from that infer dispositional properties of the situation-episode participants. They categorize entities that move themselves (e.g., human hands) as potential causal agents and project patienthood onto inert entities (e.g., bean bags). For example, when the infants construe a moving object as an inert entity (which has no control over itself and others), they look for an external explanation for the motion. They do not do so when they take a moving object as self-regulating. This is precisely the behavior that an evaluation based on control-asymmetry predicts (see also Lai and Piñango, 2019 for discussion).

^22^Part-whole relations are low *control-asymmetry* because as “the part” is contained by “the whole”—making “the whole” a controller—“the whole,” by definition, depends on “the part” for its existence/functionality—making “the part” a controller. This situation renders part-whole relations control *symmetrical*: they demand that both entities be controller and controlee.

^23^A reviewer asks whether the model is restricted to control-asymmetry and connectedness or open to more aspects, that might be found to be relevant given futher evidence. The model is open to further parametric constrains. As discussed here, the parameters of control-asymmetry and connectedness while fundamental are insufficient to account for all known semantico-conceptual evaluations. Some interesting possibilities are paramaters that connect to theory of mind considerations which are also “visible” through language such as evidentiality, indexicality, and distinctions among mental states.

^24^See also Piñango and Deo (2016), for a specific semantic account that capitalizes on these conceptual distinctions.

^25^['grima]: sense of discomfort from situations like someone scratching their nails on a blackboard or piercing your eye with a needle. [kafu'ne]: The act of gently running one's fingers through a loved one's hair.
